# Antibacterial and Antifungal Silver Nanoparticles with Tunable Size Embedded in Various Cellulose-Based Matrices

**DOI:** 10.3390/molecules27196680

**Published:** 2022-10-07

**Authors:** Gabriela Biliuta, Andra-Cristina Bostănaru-Iliescu, Mihai Mareș, Carla Pavlov-Enescu, Valentin Năstasă, Olga Burduniuc, Sergiu Coseri

**Affiliations:** 1 Polyaddition and Photochemistry Laboratory, “Petru Poni” Institute of Macromolecular Chemistry, 41A Grigore Ghica Voda Alley, 700487 Iasi, Romania; 2 Laboratory of Antimicrobial Chemotherapy, Faculty of Veterinary Medicine, “Ion Ionescu de la Brad” University of Life Sciences of Iasi (IULS), 8 Mihail Sadoveanu Alley, 700489 Iasi, Romania; 3 Discipline of Microbiology and Immunology, “Nicolae Testemițanu” State University of Medicine and Pharmacy, Bd. Stefan Cel Mare și Sfant 165, 2001 Chisinau, Moldova; 4 Departament of the Laboratory Diagnosis in Public Health, National Agency for Public Health, 67A Gheorghe Asachi, 2028 Chisinau, Moldova

**Keywords:** AgNPs, cellulose derivatives, antimicrobial activity, hydroxypropyl cellulose, methylcellulose, ethylcellulose, cellulose acetate

## Abstract

The aim of this study was to synthesize silver nanoparticles (AgNPs) using cellulose derivatives and to evaluate their antimicrobial potential. As effective reducing and stabilizing agents for AgNPs, cellulose derivatives, such as hydroxypropyl cellulose (HPC), methylcellulose (MC), ethylcellulose (EC), and cellulose acetate (CA), were used. Their ability to reduce silver ions as well as the size of the resulting AgNPs were compared. The formation and stability of the reduced AgNPs in the solution were monitored using UV-Vis analysis. The size, morphology, and charge of the AgNPs were evaluated. We found that, when using cellulosic derivatives, AgNPs with sizes ranging from 17 to 89 nm and different stabilities were obtained. The parameters, such as size and ζ potential indicate the stability of AgNPs, with AgNPs-CA and AgNPs-HPC being considered more stable than AgNPs-EC and AgNPs-MC since they show higher ζ potential values. In addition, the AgNPs showed antimicrobial activity against all reference strains and clinical isolates. MIC values between 0.0312 and 0.125 mM had a bactericidal effect on both Gram-positive and Gram-negative bacteria. The fungicidal effect was obtained at a MIC value of 0.125 mM. These results may provide rational support in the design of medical gauze products, including gauze pads, rolls, and sponges.

## 1. Introduction

The increasing demand for bio-based materials is gaining attention for immediate applications in biomedical fields, such as wound healing, tissue engineering, and drug delivery. Long-chain biopolymeric carbohydrate molecules are bio-based materials with huge potential in biomedical applications, exhibiting unique beneficial features of natural polymers. Polysaccharides, especially cellulose, are an important natural biopolymer due to their high abundance and wide distribution in nature, as they are a major structural component in plant cell walls [1,2,3,4].

Cellulose is a linear homopolysaccharide that is composed of repeating glucose units (C_6_H_10_O_5_)_n_ and is considered the major profuse organic material. This biodegradable polymer is mostly found in nature in its native form in the cell walls of wood, plants, some fungi, and in the membranes of epidermal cells of marine invertebrate animals [4,5,6]. Cellulose offers some advantages in relation to synthetic polymers, such as biodegradability, biocompatibility, low production cost, and low cytotoxicity.

These characteristics of cellulose offer potential as bioresorbable polymers that play a vital role in biomedical applications due to their unique ability to be completely resorbed [4]. Undoubtedly, cellulosic materials are promising for applications in biological implants, wound and burn dressing materials, medical implants, and drug delivery systems due to their excellent physical and biological properties, biodegradability, biocompatibility, and low cytotoxicity, which have already been demonstrated by different studies. 

The surface functionalization and shape of cellulose used for biomedical applications provide a useful and powerful tool to tune the interactions of biomaterials with living tissue. The utility of cellulosic materials in wound healing and organ replacement applications has already been demonstrated by several studies and also commercialized; however, more interdisciplinary research is still needed to further the development of these cellulosic materials [4,7].

Due to their many uses in medical devices, pharmaceuticals, antimicrobial agents, water purification systems, cosmetics, and the food sector, nanoparticles have attracted a great deal of attention from researchers. AgNPs in particular possess antibacterial, antiviral, antifungal, antioxidant, and anti-inflammatory properties, making them suitable for application in materials in a variety of industries [8,9,10,11]. Long-standing research has shown that many bacterial strains and other germs that often infect people are inhibited by silver [12].

AgNPs have recently played important roles in the medical field for oral and drug applications, including creams and topical ointments containing AgNPs to prevent the infection of burns and wounds and implants prepared with silver-impregnated polymers [13]. However, research is still required to identify the best process for producing AgNPs. The toxicity of AgNPs in vitro and in vivo poses a significant problem because, despite the fact that silver is often thought of as a material that is harmless, it may sometimes become harmful to mammalian cells [14]. AgNPs have been made using many different strategies and methods, including physical, chemical, and biological ones. Amongst these, chemical methods have proven to be the most useful for AgNP generation. 

A multitude of chemical reduction methods are being used to synthesize the AgNPs. Metal precursors, reducing agents, and stabilizing or capping agents, such as sodium borohydride, sodium citrate, ascorbate, and poly (ethylene glycol), are often the basic components of a chemical process. These substances are often costly and dangerous for the environment. Another drawback of these methods is that the capping agent is difficult to remove from the system. In these methods, large amounts of reducing agent are necessary to reduce the silver ions and stabilize the formed AgNPs [15,16,17,18,19].

Other methods use temperature for the silver reduction to AgNPs. For example, Abdellatif et al. studied the efficiency of different cellulosic polymers for AgNPs preparation under high temperatures [20]. It is commonly known that cellulose is highly insoluble in organic solvents in its natural state, which poses a significant obstacle to its usage in many applications. Its ether and ester derivatives have become widely used in a variety of uses as a compromise measure. Oxidation products, in addition to cellulose esters and ethers, can serve as highly valuable intermediates in a multitude of scenarios where cellulose itself cannot be employed [21,22,23,24,25,26,27].

Hence, we envisaged that the use of cellulose derivatives for the synthesis of nanoparticles could be advantageous over other processes by eliminating the laborious steps required for the nanoparticles preparation. Additionally, no extra chemical agents or harsh conditions, such as elevated pressure, high energy, or temperature, are needed in our procedure. The AgNPs are simultaneously reduced and stabilized by cellulose derivatives. In this work, we investigate how various cellulose derivatives affect the effectiveness and stability of an AgNPs formulation with antimicrobial effects in order to obtain a more consistent picture of the effect of the nature of the cellulosic substrate on the morphology and sizes of silver nanoparticles.

**Figure 1:**
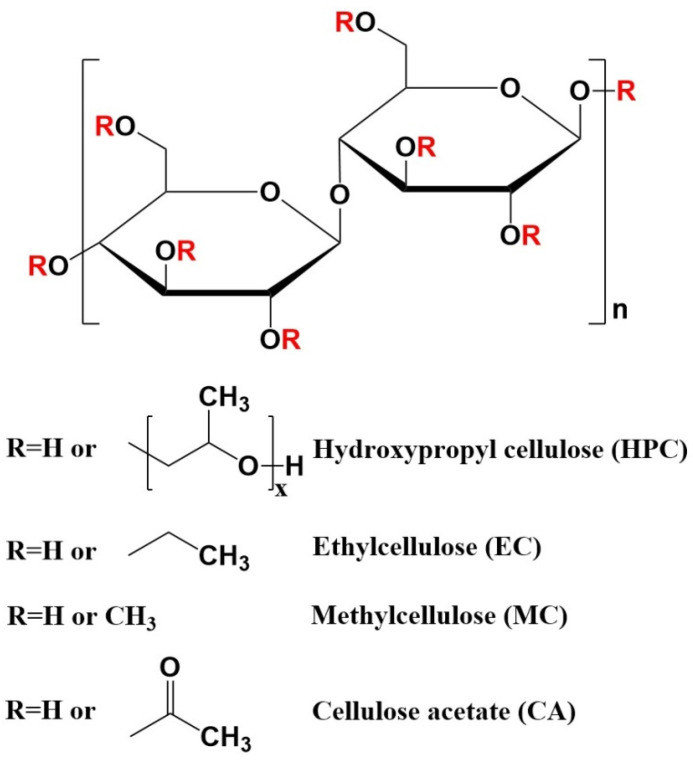
The cellulose derivatives used for the reduction and stabilization of silver nanoparticles (AgNPs).

In order to do this, AgNPs were produced using HPC, MC, EC, and CA (see Figure 1) as both reducing and capping agents in a single phase.

The resulting AgNPs are both safe and reasonably priced. The efficiency of synthesis was verified by UV-Vis spectroscopy. The size, morphology, and charge of the AgNPs were evaluated by dynamic light scattering (DLS), Scanning Transmission Electron Microscopy (STEM), and Transmission Electron Microscopy (TEM).

## 2. Results and Discussion

**Figure 2:**
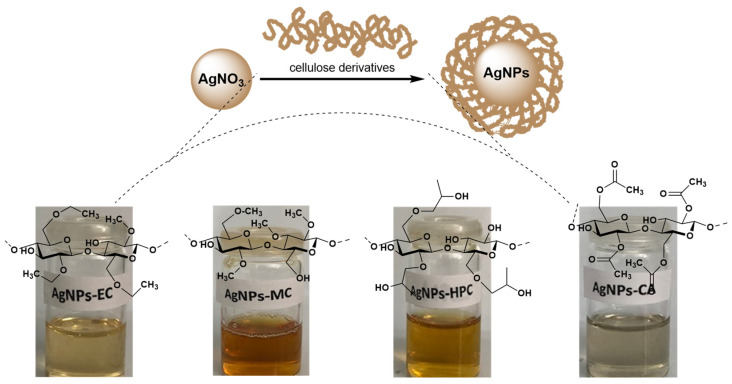
An illustration of the silver nanoparticles formation mediated by cellulose derivatives (hydroxypropyl cellulose (HPC), methylcellulose (MC), ethylcellulose (EC), and cellulose acetate (CA)).

Due to their distinct characteristics, polysaccharides are frequently employed in the manufacture of silver nanoparticles [28,29]. In the current work, the synthesis of the AgNPs was easily accomplished by combining a variety of non-ionic cellulose derivatives (CA, HPC, MC, and EC) with AgNO_3_ solution at room temperature. The silver nitrate solution changed color when 1% polymer solution was added, going from light yellow to dark brown, indicating the formation of AgNPs as a result of cellulose derivatives converting Ag^+^ ions to elemental Ag due to the surface plasmon resonance (SPR) phenomenon (Figure 2).

Practically, the silver ion interacted with cellulose derivatives by combining the two solutions to create an Ag [cellulose derivative]^+^ complex. As an intermediate precursor, the cellulose derivative converted the Ag^+^ to (Ag [cellulose derivative]). AgNPs were stabilized colloidally as a consequence of the cellulose derivatives’ negative charge capping the positively-charged groups on their surface (Ag [cellulose derivative]).

The lack of precipitation in the AgNPs-cellulose derivative systems produced under these circumstances suggests that these cellulose derivatives may serve as both a reducing agent and as a stabilizing or capping agent to prevent the formation of aggregates in the resulting AgNPs. The mechanical properties of cellulose-based nanocomposites are well documented in the specialized literature, and it is well-known that cellulose is an unbeatable material for making composites with remarkable mechanical properties, such as the tensile strength and Young’s modulus. Moreover, the introduction of silver leads to both strengthening and toughening of the cellulose matrix. Similarly, other polymeric membranes with high strength were recently reported [30].

### 2.1. UV-Vis Spectroscopy

**Figure 3:**
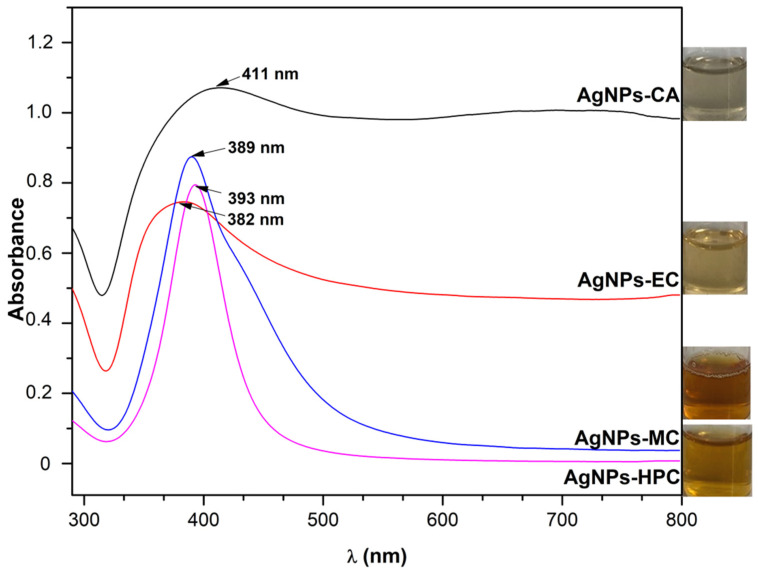
UV-Vis spectra of AgNPs synthesized from cellulose derivatives (CA—cellulose acetate, EC—ethylcellulose, MC—methylcellulose, and HPC—hydroxypropil cellulose).

In order to monitor the formation and stability of silver nanoparticles, absorption spectra of the AgNPs were obtained. In Figure 3, we present the UV-Vis spectra of AgNPs formation using the four cellulose derivatives. Depending on the kind of polymer utilized, the solutions’ colors varied. After two hours, the colorless combination of MC became dark brown, EC became light yellow, HPC became light brown, and CA turned light yellow, showing that ionic silver had been converted to silver nanoparticles as a result of the activation of the surface plasmon resonance phenomenon (SPR).

**Figure 4:**
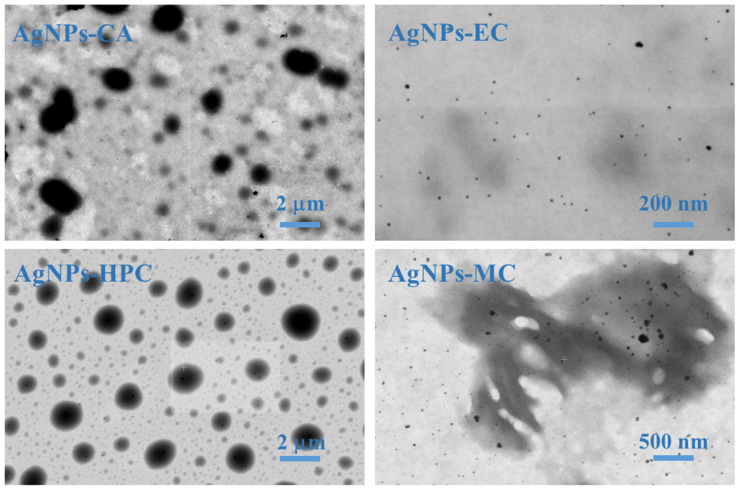
STEM images of the AgNPs synthesized from cellulose derivatives.

The effect of the reducing/stabilizing agent on the AgNPs formation is illustrated in Figure 4. It is widely known that silver nanoparticles absorb light in the visible region of the electromagnetic spectrum (380–450 nm) as a result of the SPR transition. The absorbing wavelength of AgNPs depends on the particle size, dielectric medium, and chemical surroundings as well as the nanostructure of the polymer employed to create and maintain the nanoparticles. All these affect the UV-Vis absorption spectra of the silver nanoparticles.

The SPR of AgNPs is between 382 and 411 nm (AgNPs-CA = 411 nm, AgNPs-EC = 382 nm, AgNPs-MC = 389 nm, and AgNPs-HPC = 393 nm). These wavelengths agreed with those obtained by Hajji et al., proving that AgNPs were successfully synthesized [31]. Broader peaks, as in the case of AgNPs-EC and AgNPs-CA, indicate the presence of nanoparticles in a broad range of sizes. The AgNPs were created with a symmetrical form, as shown by the fact that each preparation exhibited a single absorption peak.

### 2.2. Particle Size Analysis

The hydrodynamic diameter (Z-average), polydispersity index (PDI), and potential zeta of the synthesized AgNPs was determined by a particle size analyzer, and the results are shown in Table 1. Depending on the cellulosic derivative used, the sizes of the AgNPs samples as determined by DLC varied. Following data analysis, we observed that the particles had a substantial polydispersity index and an average particle diameter of between 119.7 ± 0.2 and 491.7 ± 0.09 nm.

Since there is always a tendency to overestimate the true size due to the strong interactions between the nanoparticles and the solvent, the sizes found cannot be taken as a real value. The mean hydrodynamic diameter affects the DLS-based measurements. Additionally, measurements of tiny aggregates in nanosuspension might alter the usual size distribution. A narrow particle size distribution is shown by the PDI values (0.263 for AgNPs-CA, 0.485 for AgNPs-EC, 0.302 for AgNPs-MC, and 0.269 for AgNPs-HPC). It is well-known that PDI estimates the homogeneity of size distribution in colloidal AgNPs solutions. PDI values higher than 0.7 denote a broad particle size distribution, with lower values indicating better homogeneity [32].

The zeta potential exhibited a negative peak, which generated high repulsion between AgNPs and therefore increased the stability of the particles for a long time. As they had greater potential values than AgNPs-EC (−8.24 ± 0.2) and AgNPs-MC (−1.93 ± 0.6), AgNPs-CA (−24.5 ± 0.5) and AgNPs-HPC (−16.2 ± 0.9) were more stable. NPs are categorized as stable if their ζ potential is greater than +10 mV or less than −10 mV [33].

### 2.3. Scanning Transmission Electron Microscopy (STEM) and Transmission Electron Microscopy (TEM)

AgNPs appear to be predominantly spherical, tiny, discrete entities in STEM images either as independent particles, aggregated particles, or as particles with different morphologies. Comparing the images, AgNPs-EC, AgNPs-CA, and AgNPs-HPC formed the largest uniform non-aggregated particles, while AgNPs-MC formed the largest, most aggregated nanoparticles (Figure 4).

The interactions between Ag^+^ and the C-O or OH groups on the coated polymers determine the NP shapes. Through these interactions, the metallic cation mobility is reduced, thereby, inhibiting the formation of large particles and stabilizing the resulting AgNPs.

**Figure 5:**
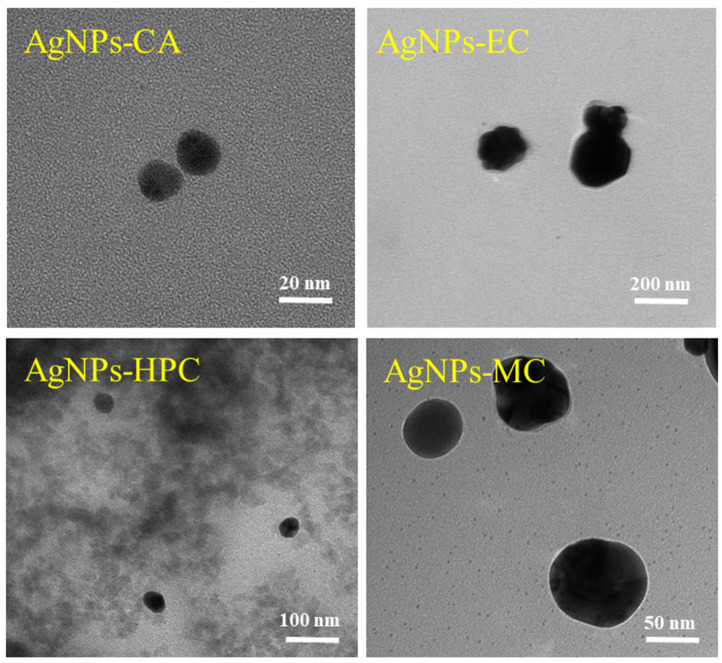
TEM micrographs of AgNPs prepared using different cellulose derivatives.

AgNPs produced in the presence of cellulose derivatives were examined using Transmission Electron Microscopy. The sizes and forms of the nanoparticles were determined using the TEM method. The TEM results demonstrated that the type of cellulose derivatives used has an impact on the average size of the produced AgNPs. The TEM images of the samples are shown in Figure 5, and the sizes of the nanoparticles are shown in Table 2. The AgNPs formed larger aggregates in the case of AgNPs-EC (89 ± 1.3 nm), AgNPs-HPC (62 ± 3.6 nm), and AgNPs-MC (54 ± 11.6 nm) and small aggregates in the case of AgNPs-CA (17 ± 1.8 nm). All of the AgNPs had a spherical form. As the TEM approach only revealed the center of the NPs to be the metal core, the sizes obtained by TEM were substantially smaller than those obtained by DLC.

### 2.4. Evaluation of the Antimicrobial Activity

**Figure 6:**
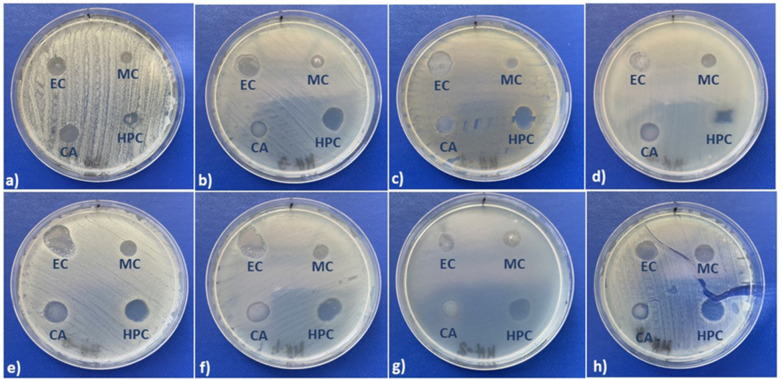
Antimicrobial activity against (**a**) *Candida albicans*, (**b**) *Escherichia coli*, (**c**) *Mycobacterium smegmatis*, (**d***) Staphylococcus aureus methicillin resistant* (MRSA), (**e**) *Pseudomonas aeruginosa*, (**f**) *Acinetobacter baumannii*, (**g**) *Enterococcus faecalis*, and (**h**) *Stenotrophomonas maltophilia*.

The antimicrobial activity was measured by the agar diffusion method, which requires the addition of the AgNPs to the culture medium pre-inoculated with the microbial suspension and the measuring of the clear zone caused by growth inhibition after 24 h of incubation (Figure 6).

**Figure 7:**
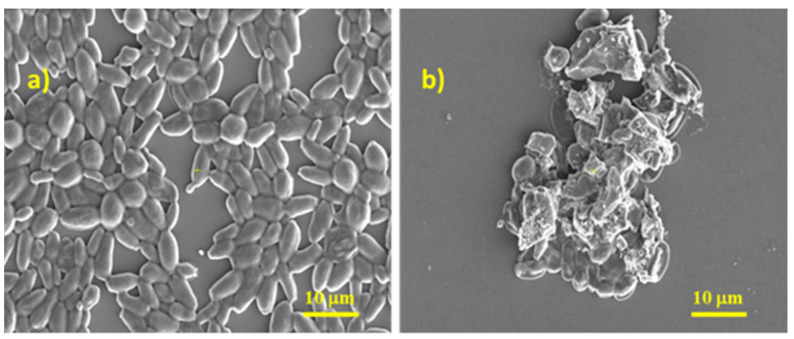
Visualization of the effects of AgNPs on *C. albicans* using advanced Scanning Electron Microscopy; (**a**) SEM micrographs of *C. albicans* without treatment, cells are well-defined, with a smooth surface and oval shaped; and (**b**) *C. albicans* after AgNPs treatment showing distortion, roughness, and disruption on the cell wall of the yeast cells.

Some data on the average inhibition diameters and images of the inhibition zones of AgNPs are given in Table 3. First, one can observe that all AgNPs generated from cellulose derivatives are able to inhibit the microbial growth of bacterial and yeast cultures placed in direct contact (Figure 7).

The antimicrobial activity of AgNPs (AgNPs-MC, AgNPs-HPC, AgNPs-CA, and AgNPs-EC) was evaluated by the microdilution method. The MIC and MFC values obtained for AgNPs against yeasts and bacteria are shown in Table 4 and Table 5. All cellulose derivatives showed the highest antimicrobial potential, with MICs values ranging between 0.0312 and 0.125 mM (silver concentration). Related to the antifungal activity of the main AgNPs, they acted similarly against *C. albicans*, *C. auris*, *C. glabrata*, *C. parapsilosis*, and *C. guilliermondii*, with a MIC value of 0.125 mM; meanwhile, *C. krusei*, *C. lusitaniae*, and *C. pelliculosa* showed a higher degree of inhibition of yeast cell growth (MIC value of 0.0626 mM).

Compared with fungi, the investigated AgNPs against bacteria showed better microbial activity, with MICs between 0.0312 and 0.125 mM. AgNPs also showed the highest-level antibacterial activity against *E. faecalis*, with an MIC value of 0.0312 mM. Strong microbial activity was observed against *E. coli*, methicillin-resistant *S. aureus*, *K. pneumoniae*, and *S. maltophilia*, which possessed MICs values equal to 0.0625 mM. An MIC value of 0.125 mM was obtained for AgNPs against *M. smegmatis*, *P. aeruginosa*, and *A. baumannii*, indicating exceptional antimicrobial activity. Based on MFC/MBC values, all AgNPs generated from cellulose derivatives showed bactericidal activity against all tested strains.

Scanning Electron Microscopy (SEM) was used to detect morphological alterations of fungal cells in the presence of AgNPs (after 24 h of incubation). Control images on Candida albicans (without AgNPs) showed that the untreated cells were aggregated and well-defined, with a smooth cell surface, an oval-shaped morphology, and more budding cells (Figure 7a). SEM images of the treated AgNPs on C. albicans showed that cells presented deep wrinkles and deformity, indicating cell-wall damage by disruption of the outer surface of the cell wall (Figure 7b).

## 3. Materials and Methods

### 3.1. Materials

Silver nitrate (AgNO_3_, ACS reagent, ≥99.0%), hydroxypropyl cellulose (HPC, 80 kDa), methylcellulose (MC, viscosity 4.000 cP), ethylcellulose (EC, 48.8% ethoxyl basis, viscosity 310 cP), cellulose acetate (CA, average Mn ~ 50.000) and dimethyl sulfoxide (DMSO, analytical grade, ≥99.9%) were purchased from Sigma Aldrich Chemicals (St. Louis, MO, USA). All the chemicals were analytical grade reagents and were used as received without further purification.

### 3.2. Microbial Strains

The in vitro antimicrobial activity of the four types of AgNPs (AgNPs-HPC, AgNPs-MC, AgNPs-EC, and AgNPs-CA) was evaluated against representative Gram-positive and Gram-negative bacteria and fungal strains. Ten strains (*Mycobacterium smegmatis* CECT 3017, methicillin-resistant *Staphylococcus aureus* ATCC 43300, *Escherichia coli* ATCC 35218, *Pseudomonas aeruginosa* CIP 82118, *Candida albicans* ATCC 90028, *Candida auris* CBS 10913T, *Candida glabrata* ATCC 90030, *Candida parapsilosis* ATCC 22019, *Candida guilliermondii* ATCC 9533, and *Candida krusei* ATCC 6258,) and six clinical isolates (*Enterococcus faecalis* RTCC 2682, *Acinetobacter baumannii* RTCC 2286, *Klebsiella pneumoniae* RTCC 2669, *Stenotrophomonas maltophilia* RTCC 2706, *Candida lusitaniae* RTCC 1120, and *Candida pelliculosa* RTCC 1029) isolated from human infections were used as testing microorganisms (CECT: Spanish Type Culture Collection; ATCC: American Type Culture Collection; CIP: Institut Pasteur Collection; CBS: Fungal Biodiversity Centre; RTCC: Romanian Type Culture Collection). The clinical isolates were collected in hospitals from different regions of Romania and were identified using MALDI-TOF MS and DNA sequencing.

### 3.3. Silver Nanoparticle Synthesis

HPC and MC were dissolved in deionized water while EC and CA were dissolved in DMSO to create polymer solutions with a concentration of 1%. After that, silver nitrate was dissolved in deionized water or DMSO to prepare a 0.01 M solution of AgNPs. Then, 2.5 mL of 0.01 M AgNO_3_ solution was added to each polymer solution, and the final volume of the mixture solution was 12.5 mL. The solutions were then kept for 2 h under vigorous stirring. The hue of the solutions varied from clear to yellow to dark brown depending on which polymer was utilized, thereby, confirming the formation of the silver nanoparticles.

### 3.4. AgNPs Characterization

UV-Vis spectroscopy: UV-Vis spectral analysis was done with a UV-Visible JascoV-550 spectrophotometer (Analytik Jena, Jena, Germany) at a wavelength of 200–600 nm.

Scanning Transmission Electron Microscopy (STEM): With a resolution of 1 nm at 30 kV and a 20 mm Oxford EDS detector, Supra Zeiss Scanning Transmission Electron Microscopy (STEM) (FEI Company, Hillsboro, OR, USA) was used to examine the surface morphology of the synthesized AgNPs. In this case, 10 µL of the solution was pipetted onto the sample holder and left to dry before being analyzed.

Transmission Electron Microscopy (TEM): TEM images were obtained using a Hitachi High-Tech HT7700 electron microscope (Hitachi High-Technologies Corporation, Tokyo, Japan), by directly applying 10 µL of AgNPs solution to a carbon-coated copper grid and leaving the sample to dry before the TEM analysis.

Particle Size and ζ potential: The ζ potential was measured with a DLS technique (Zetasizer model Nano ZS, Malvern Instruments, Malvern, UK) with a red laser at 633 nm (He–Ne) (Malvern Panalytical, Malvern, UK). The average particle size of the synthesized AgNPs and polydispersity indices (PDIs) was also measured based on the DLS analysis. The ζ potential was calculated from the electrophoretic mobility (μ) determined at 25 °C. For kα ≫ 1 (where k is the Debye–Huckel parameter and α is the particle radius), the Smoluchowski relationship was used as shown in Equation (1):

(1) $$\zeta =\frac{\eta \mu }{\varepsilon }$$

where η is the viscosity and ε is the dielectric constant.

### 3.5. Antimicrobial Activities

Screening of the antimicrobial properties: All microorganisms were stored at −80 °C in 10% glycerol. As reproducibility depends on the log growth phase of microorganisms, fresh subcultures are used. Prior to testing, the bacteria were refreshed in Mueller–Hinton broth (Merck KGaA, Darmstadt, Germany) at 37 °C, and afterward were inoculated on Tryptone Soya Agar (Biokar, Beauvais CEDEX, France) for purity checking. Fungi were refreshed on Sabouraud Dextrose Agar with chloramphenicol (Biokar, Beauvais CEDEX, France) and were grown at 36 °C. All strains were checked during the incubation period, and microbial suspensions were prepared with these cultures in sterile saline solution to obtain turbidity optically comparable to that of the 0.5 McFarland standards (Avantor, Edmonton, AB, Canada), yielding a suspension containing approximately 1 × 10^8^ CFU mL^−1^ for all strains.

Volumes of 0.2 mL from each inoculum were spread onto Mueller–Hinton Agar for bacteria and Sabouraud Dextrose Agar for yeasts, pre-poured in Petri dishes, and the AgNPs were added after drying of the medium surface. Volumes of 10 microliters of each compound dilution were spotted onto the medium surface after its inoculation with the appropriate testing strain. To evaluate the antimicrobial properties, the inhibition of growth was measured under standard conditions after 24–48 h of incubation at 36 °C. All tests were performed in triplicate to verify the results. The diameter of the inhibition zone was measured using a caliper [34].

Determination of the minimum inhibitory concentration *(MIC):* The MIC values were assessed by the broth microdilution method using 96-well plates, according to the European Committee on Antimicrobial Susceptibility Testing Subcommittee on Antibacterial/Antifungal Susceptibility Testing guidelines and following the methodology described by Mares et al. with slight modifications [35,36,37]. Serial two-fold dilutions for all four AgNPs (AgNPs-HPC, AgNPs-MC, AgNPs-EC, and AgNPs-CA), ranging from 2 to 0.0312 mM, were prepared into Mueller Hinton Broth 2 medium 2% glucose followed by inoculation (10^5^ CFU/well).

The final concentration of DMSO did not exceed 1%. In order to assure the reliability of the tests, bacteria and yeast growth control and sterility control were used. The MIC value was considered as the lowest concentration without visual growth of the tested strains. In brief, the MFC value was assessed by inoculating 10 µL from wells that did not show growth in the MIC assay onto Mueller–Hinton Agar for bacteria and Sabouraud Dextrose Agar for yeast, followed by incubation at 37 °C for 24 h and at 30 °C for 72 h, respectively. After incubation, the minimal fungicidal concentration was considered as the lowest concentration with no visible growth or less than three colonies on the agar plates in the case of subculture (corresponding to ~99.9% killing activity). The experiment was carried out in triplicate.

Scanning Electron Microscopy: For high-resolution SEM ultrastructural observation, *Candida albicans* cells were cultured in Sabouraud Dextrose Broth for 24 h at 37 °C, under shaking at 75 rpm. The preformed cells of *Candida albicans* were then treated with or without AgNPs for an additional 24 h. After treatment with AgNPs, the cells were gently washed three times in sterile PBS and then fixed with 3% glutaraldehyde at room temperature for 1 h. After fixing the yeast cells, they were washed three times in PBS and, after washing, the samples were dehydrated through a series of ethanol concentrations of 25%, 50%, 70%, and 95% (10 min each), and finally they were kept in absolute alcohol (for 20 min).

*Candida albicans* yeast morphology was analyzed with a Verios G4 UC Scanning Electron Microscopy (Thermo Scientific, Waltham, MA, USA) equipped with an energy-dispersive X-ray spectroscopy analyzer (Octane Elect Super SDD detector (AMETEK, Tokyo, Japan). Before image capture, samples were coated with 10 nm platinum using a Leica EM ACE200 Sputter Coater (Leica Microsystems, Vienna, Austria). SEM investigations were performed in high vacuum mode using a secondary electron detector (Everhart–Thornley detector, ETD (Hillsboro, OR, USA) at an accelerating voltage of 5 kV.

## 4. Conclusions

The proposed protocol is a simple and inexpensive method for producing AgNPs that employs natural biopolymers (cellulose derivatives) avoiding the use of potentially harmful reducing agents, such as sodium borohydride (NaBH_4_) and hydroxylamine hydrochloride as well as a capping/surfactant agent, and may have benefits in both technology and medicine. The synthesized AgNPs using cellulose derivatives proved to have excellent antimicrobial activity. 

The results suggest a fairly good activity against yeasts, Gram (+) and Gram (−) bacteria of all AgNPs. This study has demonstrated high antimicrobial activity of cellulose derivatives in vitro; however, further investigations are required for proper standardization and stabilization in order to make them applicable as medical gauze products. Our results demonstrated that AgNPs had uniform antibacterial and antifungal activity against all tested isolates at relatively close concentrations.

## Figures and Tables

**Table 1:** Average size values determined by DLS, polydispersity indices, and ζ potential values of the AgNPs synthesized from cellulose derivatives.

AgNPs	Z-Average (d.nm)	Polydispersity Index (PDI)	*ζ* Potential (mV)
AgNPs-CA	432 ± 0.1	0.263	−24.5 ± 0.5
AgNPs-EC	491 ± 0.09	0.485	−8.24 ± 0.2
AgNPs-MC	348 ± 0.1	0.302	−1.93 ± 0.6
AgNPs-HPC	119.7 ± 0.2	0.269	−16.2 ± 0.9

**Table 2:** Particle size and spectral features of AgNPs determined using TEM.

AgNPs	Mean Particle Size from TEM
AgNPs-CA	17 ± 1.8 nm
AgNPs-EC	89 ± 1.3 nm
AgNPs-MC	54 ± 11.6 nm
AgNPs-HPC	62 ± 3.6 nm

**Table 3:** The diameters of the inhibition zones in the antimicrobial activity test of AgNPs against microbial strains.

Diameters of the Inhibition Zones (mm)				
Strains	AgNPs-MC	AgNPs-HPC	AgNPs-CA	AgNPs-EC
*C. albicans*	9	10	8	11
*C. auris*	10	11	9	10
*C. glabrata*	8	8	10	9
*C. parapsilosis*	9	9	11	10
*C. guiliermondii*	10	10	11	12
*C. krusei*	11	10	11	11
*C. lusitaniae*	10	10	12	11
*C. pelliculosa*	8	9	10	10
*E. coli*	7	10	8	11
*M. smegmatis*	9	10	9	13
*S. aureus*	8	10	9	12
*P. aeurginosa*	10	9	9	11
*A. baumannii*	9	11	12	13
*K. pneumoniae*	11	9	12	14
*E. faecalis*	8	9	8	9
*S. maltophilia*	11	12	10	12

**Table 4:** Antifungal activity (MIC, MFC; mM) of AgNPs.

Strains	AgNPs-MC		AgNPs-HPC		AgNPs-CA		AgNPs-EC	
	MIC	MFC	MIC	MFC	MIC	MFC	MIC	MFC
*C. albicans*	0.125	0.125	0.125	0.125	0.125	0.125	0.125	0.125
*C. auris*	0.125	0.125	0.125	0.125	0.125	0.125	0.125	0.125
*C. glabrata*	0.125	0.125	0.125	0.125	0.125	0.125	0.125	0.125
*C. parapsilosis*	0.125	0.125	0.125	0.125	0.125	0.125	0.125	0.125
*C. guilliermondii*	0.125	0.125	0.125	0.125	0.125	0.125	0.125	0.125
*C. krusei*	0.0625	0.0625	0.0625	0.0625	0.0625	0.0625	0.0625	0.0625
*C. lusitaniae*	0.0625	0.0625	0.0625	0.0625	0.0625	0.0625	0.0625	0.0625
*C. pelliculosa*	0.0625	0.125	0.0625	0.0625	0.0625	0.125	0.0625	0.125

MIC = minimum inhibitory concentration; and MFC = minimum fungicidal concentration.

**Table 5:** Antibacterial activity (MIC, MBC; mM) of AgNPs.

Strains	AgNPs-MC		AgNPs-HPC		AgNPs-CA		AgNPs-EC	
	MIC	MFC	MIC	MBC	MIC	MBC	MIC	MBC
*E. coli*	0.0625	0.0625	0.0625	0.0625	0.0625	0.0625	0.0625	0.0625
*M. smegmatis*	0.125	0.125	0.125	0.125	0.125	0.125	0.125	0.125
*S. aureus*	0.0625	0.0625	0.0625	0.0625	0.0625	0.0625	0.0625	0.0625
*P. aeurginosa*	0.125	0.125	0.125	0.125	0.125	0.125	0.125	0.125
*A. baumannii*	0.125	0.125	0.125	0.125	0.125	0.125	0.125	0.125
*K. pneumoniae*	0.0625	0.0625	0.0625	0.0625	0.0625	0.0625	0.0625	0.0625
*E. faecalis*	0.0312	0.0625	0.0312	0.0312	0.0312	0.0625	0.0312	0.0625
*S. maltophilia*	0.0625	0.0625	0.0625	0.0625	0.0625	0.0625	0.0625	0.0625

MIC = minimum inhibitory concentration; and MBC = minimum bactericidal concentration.

## Data Availability

Not applicable.

## Sample Availability

Samples of the compounds are available from the authors.

## References

[1] Culica M.E., Chibac-Scutaru A.L., Mohan T., Coseri S. (2021). Cellulose-Based Biogenic Supports, Remarkably Friendly Biomaterials for Proteins and Biomolecules. Biosens. Bioelectron..

[2] Biliuta G., Coseri S. (2019). Cellulose: A Ubiquitous Platform for Ecofriendly Metal Nanoparticles Preparation. Coord. Chem. Rev..

[3] Coseri S. (2017). Cellulose: To Depolymerize… or Not To?. Biotechnol. Adv..

[4] Seddiqi H., Oliaei E., Honarkar H., Jin J., Geonzon L.C., Bacabac R.G., Klein-Nulend J. (2021). Cellulose and Its Derivatives: Towards Biomedical Applications. Cellulose.

[5] French A.D. (2017). Glucose, Not Cellobiose, Is the Repeating Unit of Cellulose and Why That Is Important. Cellulose.

[6] Lahiri D., Nag M., Dutta B., Dey A., Sarkar T., Pati S., Edinur H.A., Kari Z.A., Noor N.H.M., Ray R.R. (2021). Bacterial Cellulose: Production, Characterization, and Application as Antimicrobial Agent. Int. J. Mol. Sci..

[7] Pal S., Nisi R., Stoppa M., Licciulli A. (2017). Silver-Functionalized Bacterial Cellulose as Antibacterial Membrane for Wound-Healing Applications. ACS Omega.

[8] Shanmuganathan R., Karuppusamy I., Saravanan M., Muthukumar H., Ponnuchamy K., Ramkumar V.S., Pugazhendhi A. (2019). Synthesis of Silver Nanoparticles and Their Biomedical Applications - A Comprehensive Review. Curr. Pharm. Des..

[9] Gurunathan S., Park J.H., Han J.W., Kim J.H. (2015). Comparative Assessment of the Apoptotic Potential of Silver Nanoparticles Synthesized by Bacillus Tequilensis and Calocybe Indica in MDA-MB-231 Human Breast Cancer Cells: Targeting P53 for Anticancer Therapy. Int. J. Nanomedicine.

[10] Deeksha B., Sadanand V., Hariram N., Rajulu A.V. (2021). Preparation and Properties of Cellulose Nanocomposite Fabrics with in situ Generated Silver Nanoparticles by Bioreduction Method. J. Bioresour. Bioprod..

[11] Yorseng K., Siengchin S., Ashok B., Rajuluc A.V. (2020). Nanocomposite egg shell powder with in situ generated silver nanoparticles using inherent collagen as reducing agent. J. Bioresour. Bioprod..

[12] Jiang H., Manolache S., Wong A.C.L., Denes F.S. (2004). Plasma-Enhanced Deposition of Silver Nanoparticles onto Polymer and Metal Surfaces for the Generation of Antimicrobial Characteristics. J. Appl. Polym. Sci..

[13] Becker R.O. (1999). Silver Ions in the Treatment of Local Infections. Met. Based. Drugs.

[14] Alexander J.W. (2009). History of the Medical Use of Silver. Surg. Infect..

[15] Pastoriza-Santos I., Liz-Marzán L.M. (2002). Formation of PVP-Protected Metal Nanoparticles in DMF. Langmuir.

[16] Chen M., Wang L.Y., Han J.T., Zhang J.Y., Li Z.Y., Qian D.J. (2006). Preparation and Study of Polyacryamide-Stabilized Silver Nanoparticles through a One-Pot Process. J. Phys. Chem. B.

[17] Kuo P.L., Chen W.F. (2003). Formation of Silver Nanoparticles under Structured Amino Groups in Pseudo-Dendritic Poly(Allylamine) Derivatives. J. Phys. Chem. B.

[18] Zou X., Bao H., Guo H., Zhang L., Qi L., Jiang J., Niu L., Dong S. (2006). Mercaptoethane Sulfonate Protected, Water-Soluble Gold and Silver Nanoparticles: Syntheses, Characterization and Their Building Multilayer Films with Polyaniline via Ion–Dipole Interactions. J. Colloid Interface Sci..

[19] Popa M., Pradell T., Crespo D., Calderón-Moreno J.M. (2007). Stable Silver Colloidal Dispersions Using Short Chain Polyethylene Glycol. Colloids Surf. A Physicochem. Eng. Asp..

[20] Abdellatif A.A.H., Alturki H.N.H., Tawfeek H.M. (2021). Different Cellulosic Polymers for Synthesizing Silver Nanoparticles with Antioxidant and Antibacterial Activities. Sci. Rep..

[21] Coseri S., Biliuta G., Simionescu B.C. (2018). Selective Oxidation of Cellulose, Mediated by: *N* -Hydroxyphthalimide, under a Metal-Free Environment. Polym. Chem..

[22] Baron R.I., Bercea M., Avadanei M., Lisa G., Biliuta G., Coseri S. (2019). Green Route for the Fabrication of Self-Healable Hydrogels Based on Tricarboxy Cellulose and Poly(Vinyl Alcohol). Int. J. Biol. Macromol..

[23] Culica M.E., Biliuta G., Rotaru R., Lisa G., Baron R.I., Coseri S. (2019). New Electromagnetic Shielding Materials Based on Viscose-Carbon Nanotubes Composites. Polym. Eng. Sci..

[24] Nica I., Zaharia C., Baron R.I., Coseri S., Suteu D. (2020). Adsorptive Materials Based on Cellulose: Preparation, Characterization and Application for Copper Ions Retention. Cellul. Chem. Technol..

[25] Culica M.E., Chibac-Scutaru A.L., Melinte V., Coseri S. (2020). Cellulose Acetate Incorporating Organically Functionalized CeO2 NPs: Efficient Materials for UV Filtering Applications. Materials.

[26] Culica M.E., Avadanei M., Baron R.I., Chibac-Scutaru A.L., Asandulesa M., Biliuta G., Lisa G., Coseri S. (2020). The Source of Conductivity and Proton Dynamics Study in TEMPO-Oxidized Cellulose Doped with Various Heterocyclic Molecules. Cellulose.

[27] Baron R.I., Coseri S. (2020). Preparation of Water-Soluble Cellulose Derivatives Using TEMPO Radical-Mediated Oxidation at Extended Reaction Time. React. Funct. Polym..

[28] Burduniuc O., Bostanaru A.C., Mares M., Biliuta G., Coseri S. (2021). Synthesis, Characterization, and Antifungal Activity of Silver Nanoparticles Embedded in Pullulan Matrices. Materials.

[29] Coseri S., Spatareanu A., Sacarescu L., Rimbu C., Suteu D., Spirk S., Harabagiu V. (2015). Green Synthesis of the Silver Nanoparticles Mediated by Pullulan and 6-Carboxypullulan. Carbohydr. Polym..

[30] Xu T.C., Wang C.S., Hu Z.Y., Zheng J.J., Jiang S.H., He S.J., Hou H.Q. (2022). High Strength and Stable Proton Exchange Membrane Based on Perfluorosulfonic Acid/Polybenzimidazole. Chin. J. Polym. Sci..

[31] Hajji S., Salem R.B.S.B., Hamdi M., Jellouli K., Ayadi W., Nasri M., Boufi S. (2017). Nanocomposite Films Based on Chitosan–Poly(Vinyl Alcohol) and Silver Nanoparticles with High Antibacterial and Antioxidant Activities. Process Saf. Environ. Prot..

[32] Nasiriboroumand M., Montazer M., Barani H. (2018). Preparation and Characterization of Biocompatible Silver Nanoparticles Using Pomegranate Peel Extract. J. Photochem. Photobiol. B Biol..

[33] Barhoum A., García-Betancourt M.L., Rahier H., Van Assche G. (2018). Physicochemical Characterization of Nanomaterials: Polymorph, Composition, Wettability, and Thermal Stability. Emerging Applications of Nanoparticles and Architecture Nanostructures.

[34] The European Committee on Antimicrobial Susceptibility Testing Routine and Extended Internal Quality Control for MIC Determination and Disk Diffusion as Recommended by EUCAST. http://www.eucast.org.

[35] Weinstein M.P., Patel J.B. (2018). Methods for Dilution Antimicrobial Susceptibility Tests for Bacteria That Grow Aerobically: M07-A11.

[36] Golus J., Sawicki R., Widelski J., Ginalska G. (2016). The Agar Microdilution Method – a New Method for Antimicrobial Susceptibility Testing for Essential Oils and Plant Extracts. J. Appl. Microbiol..

[37] Rodriguez-Tudela J.L., Arendrup M.C., Barchiesi F., Bille J., Chryssanthou E., Cuenca-Estrella M., Dannaoui E., Denning D.W., Donnelly J.P., Dromer F. (2008). EUCAST Definitive Document EDef 7.1: Method for the Determination of Broth Dilution MICs of Antifungal Agents for Fermentative Yeasts. Clin. Microbiol. Infect..

